# Hyperdense Artery Sign and Clinical Outcomes After Endovascular Treatment in Acute Basilar Artery Occlusion

**DOI:** 10.3389/fneur.2022.830705

**Published:** 2022-04-25

**Authors:** Jinrong Hu, Wencheng He, Bo Zheng, Fang Huang, Kefeng Lv, Jiasheng Liao, Zhao Chen, He Jiang, Kuiyun Wang, Hongjun Wang, Yang Lei, Jiachuan Liao, Hongfei Sang, Shuai Liu, Weidong Luo, Ruidi Sun, Jie Yang, Jiacheng Huang, Jiaxing Song, Fengli Li, Wenjie Zi, Chen Long, Qingwu Yang

**Affiliations:** ^1^Department of Neurology, Xinqiao Hospital and The Second Affiliated Hospital, Army Medical University (Third Military Medical University), Chongqing, China; ^2^Department of Neurology, Guiping People's Hospital, Guiping, China; ^3^Department of Neurology, Yaan Peoples's Hospital, Yaan, China; ^4^Department of Neurology, People's Hospital of Yuxi City, Yuxi, China; ^5^Department of Neurology, Dongguan People's Hospital of Southern Medical University, Dongguan, China; ^6^Department of Neurology, Suining No.1 People's Hospital, Suining, China; ^7^Department of Neurology, The First People's Hospital of Neijiang, Neijiang, China; ^8^Department of Neurology, The Jintang First People's Hospital, Jintang, China; ^9^Department of Neurology, Fengdu People's Hospital, Fengdu, China; ^10^Department of Neurology, Wulong District People's Hospital, Chongqing, China; ^11^Department of Neurology, Santai County People's Hospital of North Sichuan Medical College, Santai, China; ^12^Department of Emergency, Xiangtan Central Hospital, Chongqing, China

**Keywords:** hyperdense basilar artery sign, basilar artery occlusion, endovascular treatment, favorable outcome, recanalization

## Abstract

**Background:**

This study aimed to investigate the association between the hyperdense basilar artery sign (HBAS) on non-enhanced computed tomography (CT) and clinical outcomes in patients with acute basilar artery occlusion (BAO) who underwent endovascular treatment (EVT).

**Methods:**

Eligible patients who underwent EVT due to acute BAO between January 2014 and May 2019 were divided into two groups based on HBAS. HBAS was assessed by two neuroradiologists using five grades on nonenhanced CT. The primary outcome was a favorable functional outcome (defined as a modified Rankin Scale [mRS] of 0–3) at 90 days. Secondary outcomes included successful recanalization and mortality within 90 days.

**Results:**

Among 829 patients with BAO as assessed with CT angiography, magnetic resonance angiography, or digital subtraction angiography, 643 patients were treated with EVT. Of these, 51.32% (330/643) had HBAS. Patients with HBAS were older and had more severe neurological deficits and a higher frequency of atrial fibrillation than those without HBAS. There was no significant difference in favorable outcome (adjusted odds ratio [aOR]: 1.354, 95% confidence interval [CI]: 0.906–2.024; *p* = 0.14), successful recanalization (aOR: 0.926, 95% CI: 0.616-−1.393; *p* = 0.71), and mortality (aOR: 1.193, 95% CI: 0.839–1.695; *p* = 0.33) between patients with or without HBAS. Subgroup analysis showed that the HBAS predicted a favorable outcome in patients aged <60 years (aOR: 2.574, 95% CI: 1.234–5.368; *p* = 0.01) and patients with vertebral artery-V4 segment occlusion (aOR: 3.738, 95% CI: 1.212–11.530; *p* = 0.02). In patients with HBAS, the baseline National Institutes of Health Stroke Scale (NIHSS) score, posterior circulation–Acute Stroke Prognosis Early Computed Tomography Score (pc-ASPECTS), and stent retriever were associated with successful recanalization.

**Conclusions:**

Our study did not find a significant association between HBAS and favorable outcomes and successful recanalization in patients with BAO who underwent EVT. Moreover, large prospective studies are warranted to further investigate this relationship.

## Introduction

Acute basilar artery occlusion (BAO) is a disastrous type of ischemic stroke (1, 2) as nearly 70% of patients die or survive with severe disabilities (3). Recently, mechanical thrombectomy using a stent retriever or large-bore aspiration catheter has been identified as an effective and safe treatment for BAO in posterior circulation and could result in a higher rate of desirable outcomes (4).

Hyperdense artery sign (HAS) is an early sign of acute cerebral artery occlusion observed on non-enhanced computed tomography (CT) (5–7). HAS was considered to be associated with thrombus composition. Erythrocytes forming the predominant component of some thrombi present as a high-density area in the occluded artery, thus correlating histopathologically with HAS (8–10). Conversely, thrombi predominantly composed of fibrin/platelets do not present as a high-density area on nonenhanced CT, and are thus a negative HAS. In the anterior circulation, Froehler et al. (11) and Mokin et al. (12) have reported that high-density thrombi predicted a higher rate of successful recanalization after thrombectomy in patients with large vessel occlusion. In these cases, HAS could guide the strategy of recanalization to increase the first-pass effect and decrease procedural complications. Mohammaden et al. (13) indicated that patients with HAS could have a better response to stent retriever in anterior circulation ischemic strokes, possibly because the erythrocytes are deformable and soft, thus simplifying recanalization of the occluded vessel. HAS has been observed in 35.4 to 71% of patients with posterior circulation ischemic stroke and was found to be associated with more severe initial neurological deficits, larger infarction territory, and poorer functional outcomes in the era of intravenous thrombolysis (5, 6, 14, 15). Shu et al. (16) in a series of 51 patients with BAO who underwent endovascular treatment (EVT), also demonstrated that the presence of a high-density thrombus independently predicted a favorable outcome at 90 days, although the association with recanalization was not significant. However, this study was limited to small sample size and old-generation devices. In the new era of mechanical thrombectomy, the association of HAS with clinical outcomes after EVT in BAO patients was unclear and needed to be confirmed further.

In this study, we analyzed the association of clinical outcomes and the presence of a hyperdense basilar artery sign (HBAS) identified in patients with BAO within 24 h and who underwent EVT. Furthermore, the predictors of successful recanalization and favorable outcomes were also studied in patients with HBAS.

## Methods

### Patient Selection

We analyzed patients with acute BAO from 47 comprehensive stroke centers in the BASILAR study, which was a prospective multicenter registry in China, from January 2014 to May 2019. A previous study outlined the inclusion and exclusion criteria (4). The ethics committee at the participating centers approved the study protocol. In addition, informed consent was obtained from all patients or their legal representatives.

### Data Collection

Medical history, clinical presentation, and laboratory findings were obtained as part of the patients' workup. We also collected the time of posterior circulation stroke, stroke severity, and neurological deficits on admission; pretreatment and post-treatment imaging findings, type of treatment, complications, and stroke etiology. Stroke severity on admission was categorized as severe or mild-to-moderate. Patients in a coma with tetraplegia or in a locked-in state were defined as having a severe stroke, whereas any deficit that was less than severe was defined as mild-to-moderate stroke. Neurological deficits on admission were estimated using the National Institutes of Health Stroke Scale (NIHSS) (17).

### Neuroimaging Review

Imaging data were individually reviewed by two neuroradiologists (Drs. Chen and Qiu) with over 10 years of experience in an independent core laboratory. Both were blinded to the clinical information. We assessed the ischemic change at baseline using the posterior circulation–Acute Stroke Prognosis Early Computed Tomography Score (pc-ASPECTS) (18). Collateral status was assessed using the American Society of Interventional and Therapeutic Neuroradiology/Society of Interventional Radiology (ASITN/SIR) collateral grading system and divided into ASITN/SIR grades 0–1, 2, and 3–4 (19). The Trial of ORG10172 in Acute Stroke Treatment (TOAST) classification was used to determine the etiology of stroke. In addition, the occlusion sites of the basilar artery were subdivided into distal, middle, proximal, and vertebral artery-V4 (VA-V4). In particular, the definition of the distal vertebral artery (VA-V4 segment) occlusion included in this study refers to the V4 segment occlusion of the isolated or dominant vertebral artery.

**Figure 1:**
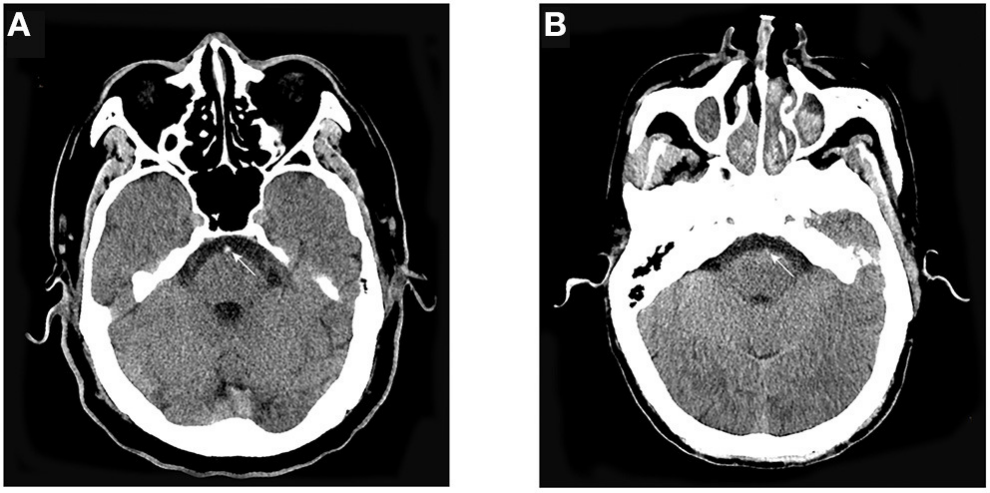
Representative cases. **(A)** A 62-year-old man presented acute onset of slurred speech and progressed to loss of consciousness. The basilar artery appears hyperdense on nonenhanced CT. **(B)** A 71-year-old man presented acute onset of dizziness and slurred speech. The basilar artery did not appear hyperdense on nonenhanced CT.

Patients underwent nonenhanced CT performed with 3 to 5-mm section thickness. We analyzed the presence of a dense vessel sign on nonenhanced CT using the method reported by Goldmakher et al.^6^ The presence of HBAS was evaluated based on a 5-point scale as follows: 5, definitely present; 4, probably present; 3, uncertainty; 2, probably absent; 1, definitely absent. For this analysis, HBAS was defined as a score of 4 or 5 points (example in Figure 1). Disagreements between the two reviewers were resolved by a third neuroradiologist (Dr. Zi).

### Outcome Measures

The primary outcome was a favorable functional outcome at 90 days, which was defined as a modified Rankin scale (mRS) score of 0–3. Secondary outcomes included successful recanalization and mortality within 90 days. Recanalization was evaluated using the modified thrombolysis in cerebral infarction (mTICI) scale. An mTICI grade of 2b to 3 was considered successful recanalization at the end of the intervention. Other outcomes included good outcomes (defined as mRS 0–2) at 90 days and symptomatic intracerebral hemorrhage (sICH) within 48 h. Intracerebral hemorrhage on follow-up imaging was assessed according to the Heidelberg Bleeding Classification (20). We defined sICH as an increase by ≥4 points of NIHSS, or an increase by ≥2 points in one NIHSS category, or requiring intubation, hemicraniectomy, and external ventricular drain placement due to clinical deterioration.

### Statistical Analysis

Baseline characteristics were analyzed between the negative and positive HBAS groups using the Mann–Whitney *U*-test for continuous variables, while Pearson's chi-square test or Fisher's exact test was used for categorical variables. The baseline characteristics were presented as median and interquartile range or as frequency and percentage (%). Statistical analysis was performed using SPSS version 26.0 (IBM, Armonk, NY). Statistical significance was set at a two-sided *p* < 0.05 and was used throughout the study.

The association of clinical or radiological outcomes with HBAS was analyzed using multivariate logistic regression and linear regression models, as appropriate. For each outcome variable, we calculated the unadjusted and adjusted odds ratios (aORs). The covariates included baseline variables and the parameters of the surgical procedure, which were selected according to baseline differences and other studies. Next, subgroup analysis to evaluate the association of HBAS with a favorable outcome was performed by adjusting the baseline variables (age, hypertension, diabetes, NIHSS, pc-ASPECTS, stroke etiology, occlusion sites, onset to treatment time (OTT), and ASITN/SIR). In addition, in patients with HBAS, the predictors of a favorable outcome and successful recanalization were analyzed. These predictors were selected from Supplementary Table 1 and Supplementary Table 2 in the univariate analysis at a *p* < 0.1 level and from common predictors in large clinical studies.

## Results

### Baseline Characteristics

**Figure 2:**
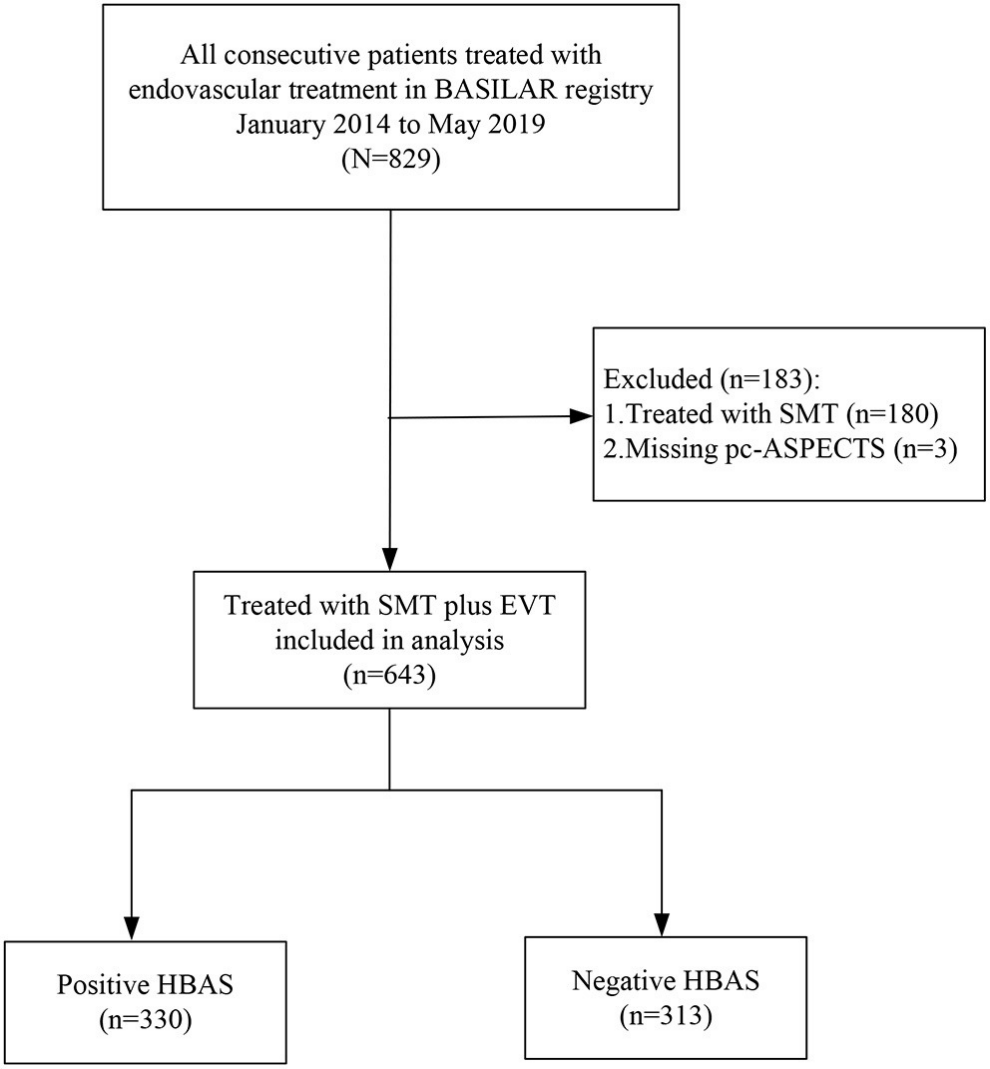
Flowchart of included patients. BASILAR indicated Endovascular Treatment for Acute Basilar Artery Occlusion Study; SMT, denoted standard medical treatment; EVT, endovascular treatment; pc-ASPECTS, displayed Posterior Circulation-Alberta Stroke Program Early; HBAS, hyperdense basilar artery sign.

In the BASILAR study, 829 patients with acute BAO were enrolled within 24 h of the estimated occlusion time. Therein, 6 patients without admission CT and 180 patients treated only with standard medical therapy were excluded (in Figure 2). Among the eligible 643 patients treated with EVT, 330 patients were noted with HBAS (76.1% men; median age, 65 [59–74] years). The baseline characteristics of the cohort based on the presence of HBAS are presented in Table 1.

**Table 1:** Baseline characteristics.

	**Negative HBAS**	**Positive HBAS**	***P-*value**
	**(*n* = 313)**	**(*n* = 330)**	
Age, median (IQR), y	63(55–71)	65(59–74)	0.002
≥65, *n* (%)	138(44.1)	180(54.5)	0.008
Male, *n* (%)	228(72.8)	251(76.1)	0.35
Baseline NIHSS score, median (IQR)	25(15–32)	28(18–34)	0.004
**Neurological deficit at admission**, ***n*** **(%)**			
Mild to moderate	85(27.2)	55(16.7)	0.001
Severe	228(72.8)	275(83.3)	
Baseline pc-ASPECTS, median (IQR)	8(7–9)	8(7–)	0.43
≥8, *n* (%)	198(63.3)	192(58.2)	0.19
ASITN/SIR, median (IQR)	1(1–2)	1(1–2)	0.07
**ASITN/SIR grade**, ***n*** **(%)**			0.06
0–1	181(57.8)	205(62.1)	
2	82(26.2)	93(28.2)	
3–4	50(16)	32(9.7)	
**Risk factors**, ***n*** **(%)**			
Hypertension	218(69.6)	231(70.0)	0.92
Diabetes	73(23.3)	75(22.7)	0.86
Atrial fibrillation	52(16.6)	84(25.5)	0.006
Smoking	117(37.4)	118(35.8)	0.67
**Stroke etiology**, ***n*** **(%)**			0.21
LAA	213(68.1)	203(61.5)	
CE	74(23.6)	97(29.4)	
Other	26(8.3)	30(9.1)	
**Occlusion cites**, ***n*** **(%)**			0.36
BA distal	101(32.3)	119(36.1)	
BA middle	93(29.7)	101(30.6)	
BA proximal	51(16.3)	56(17)	
VA-V4	68(21.7)	54(16.4)	
**Time metrics, median (IQR), min**			
OTR	442(333–645)	440(321.5–624.5)	0.96
DTP	134(92–215)	129(80.5–188.5)	0.10
DTR	252(186–350)	244(184–335)	0.20
PTR	105(72–146)	104(70–152)	0.88

*HBAS, hyperdense basilar artery sign; IQR, interquartile range; NIHSS, National Institutes of Health Stroke Scale; pc-ASPECTS, posterior circulation Acute Stroke Prognosis Early Computed Tomography Score; ASITN/SR, American Society of interventional and Therapeutic Neuroradiology/Society of interventional Radiology System; LAA, large artery atherosclerosis; CE, cardioembolism; BA, basilar artery; VA-V4, vertebral artery-V4 segment; OTR, onset to recanalization time; DTP, door to puncture time; DTR, door to recanalization time; PTR, puncture to recanalization time*.

The HBAS group had a higher median age (65 [59–74] years vs. 63 [55–71] years, *p* = 0.002) and a higher proportion of atrial fibrillation (25.5 vs. 16.6%, *p* = 0.006) compared to the negative HBAS group. Patients with HBAS also had higher NIHSS scores (median, 28 [18–34] vs. 25 [15–32], *p* = 0.004) on admission and a greater proportion of severe neurological deficits (83.3 vs. 72.8%, *p* = 0.001).

### Association of HBAS With Clinical Outcomes

Among the 330 patients with HBAS, 106 (32.1%) achieved favorable outcomes, 263 (79.7%) achieved successful recanalization, and 164 (49.7%) patients died. A total of 24 patients (7.4%) experienced sICH within 48 h. Moreover, a first-pass effect was noted in 129 patients (39.1%).

**Figure 3:**
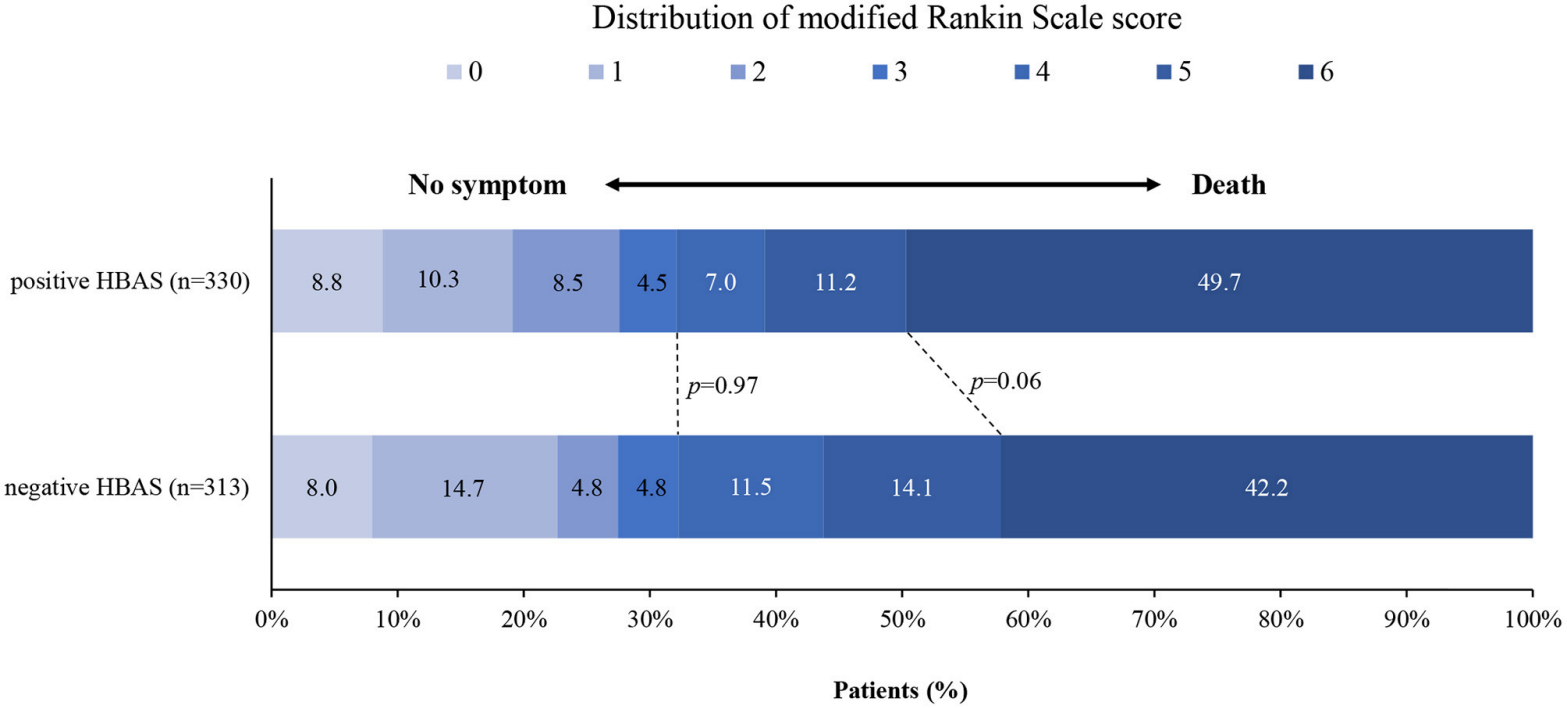
Distribution of the modified Rankin scale (mRS) score at 90 days in patients with negative and positive hyperdense basilar artery sign (HBAS) who underwent EVT. The distribution showed that when compared with negative HBAS, there were no significant differences in favorable outcomes and mortality for patients with HBAS.

Figure 3 shows the distribution of the mRS values in the HBAS and negative HBAS groups. There were no significant differences in favorable outcomes (32.1% vs. 32.3%, *p* = 0.97) and mortality (49.7% vs. 42.2%, *p* = 0.06) between the two groups. In the logistic regression analyses, HBAS was not associated with favorable outcomes (aOR: 1.354, 95% CI: 0.906–2.024; *p* = 0.14), successful recanalization (aOR: 0.926, 95% CI: 0.616–1.393; *p* = 0.71), sICH (aOR: 0.990, 95% CI: 0.528–1.856; *p* = 0.98), and mortality (aOR: 1.193, 95% CI: 0.839–1.695; *p* = 0.33) after adjusting for age, atrial fibrillation, baseline NIHSS score, pc-ASPECTS, stroke etiology, and occlusion sites (Table 2). Sensitive analyses in additional adjustment for onset to puncture time and number of attempts also showed that HBAS was not associated with clinical outcomes after EVT.

**Table 2:** The association of HBAS with clinical and radiological outcomes.

	**Negative**	**Positive**	**Unadjusted OR (CI 95%)**,	**Adjusted ^†^OR (CI 95%)**,	**Adjusted^‡^ OR (CI 95%)**,
	**HBAS**	**HBAS**	***p-*value**	***p*-value**	***p*-value**
**Clinical outcomes**, ***n*** **(%)**					
mRS 0–2 at 90-day	86(27.5)	91(27.6)	1.005(0.711–1.421), 0.98^a^	1.440(0.942–2.201), 0.09	1.378(0.879–2.160), 0.16
mRS 0-3 at 90-day	101(32.3)	106(32.1)	0.993(0.713–1.383), 0.97^a^	1.354(0.906–2.024), 0.14	1.322(0.866–2.019), 0.20
Mortality within 90-day	132(42.2)	164(49.7)	1.355(0.992–1.849), 0.06^a^	1.193(0.839–1.695), 0.33	1.217(0.839–1.763), 0.30
sICH within 48-hour	21(6.8)	24(7.4)	1.105(0.602–2.028), 0.75^a^	0.990(0.528–1.856), 0.98	0.845(0.426–1.677), 0.63
**Radiological outcomes**					
mTICI ≥2b, *n* (%)	256(81.8)	263(79.7)	0.874(0.590–1.295), 0.50^a^	0.926(0.616–1.393), 0.71	0.857(0.546–1.344), 0.50
mTICI ≥2c, *n* (%)	196(62.6)	197(59.7)	0.884(0.644–1.215), 0.45^a^	0.935(0.673–1.298), 0.69	0.894(0.631–1.267), 0.53
First pass effect, median (IQR)	132(42.2)	129(39.1)	0.880(0.642–1.206), 0.43^a^	0.925(0.669–1.281), 0.64	0.993(0.675–1.459), 0.97
The number of passes	1(1–2)	2(1–2)	0.145(-0.041 to 0.331), 0.127^b^	0.086(-0.102 to 0.273), 0.37	0.089(-0.099 to 0.277), 0.353
with stent retriever, median (IQR)					
PTR with stent retriever	103(71.5–141)	103(71–151)	0.315(−10.202t to 10.833), 0.95^b^	2.232(−8.300 to 12.763), 0.68	1.928(−8.180 to 12.036), 0.71
in minutes, median (IQR)					

† *adjusted variables: age, atrial fibrillation, baseline NIHSS, baseline pc-ASPECTS, stroke of etiology, occlusion sites*.

‡ *adjusted variables: additional onset to puncture time and number of attempts*.

a *The odds ratios were estimated from a binary logistic regression model*.

b *The β values were estimated from a multivariable linear regression model*.

*HBAS, hyperdense basilar artery sign; OR, odds ratio; CI, confidence interval; IQR, interquartile range; mTICI, modified thrombolysis in cerebral infarction; sICH, symptomatic intracranial hemorrhage; PTR, puncture to recanalization times*.

### Subgroup Analyses

**Figure 4:**
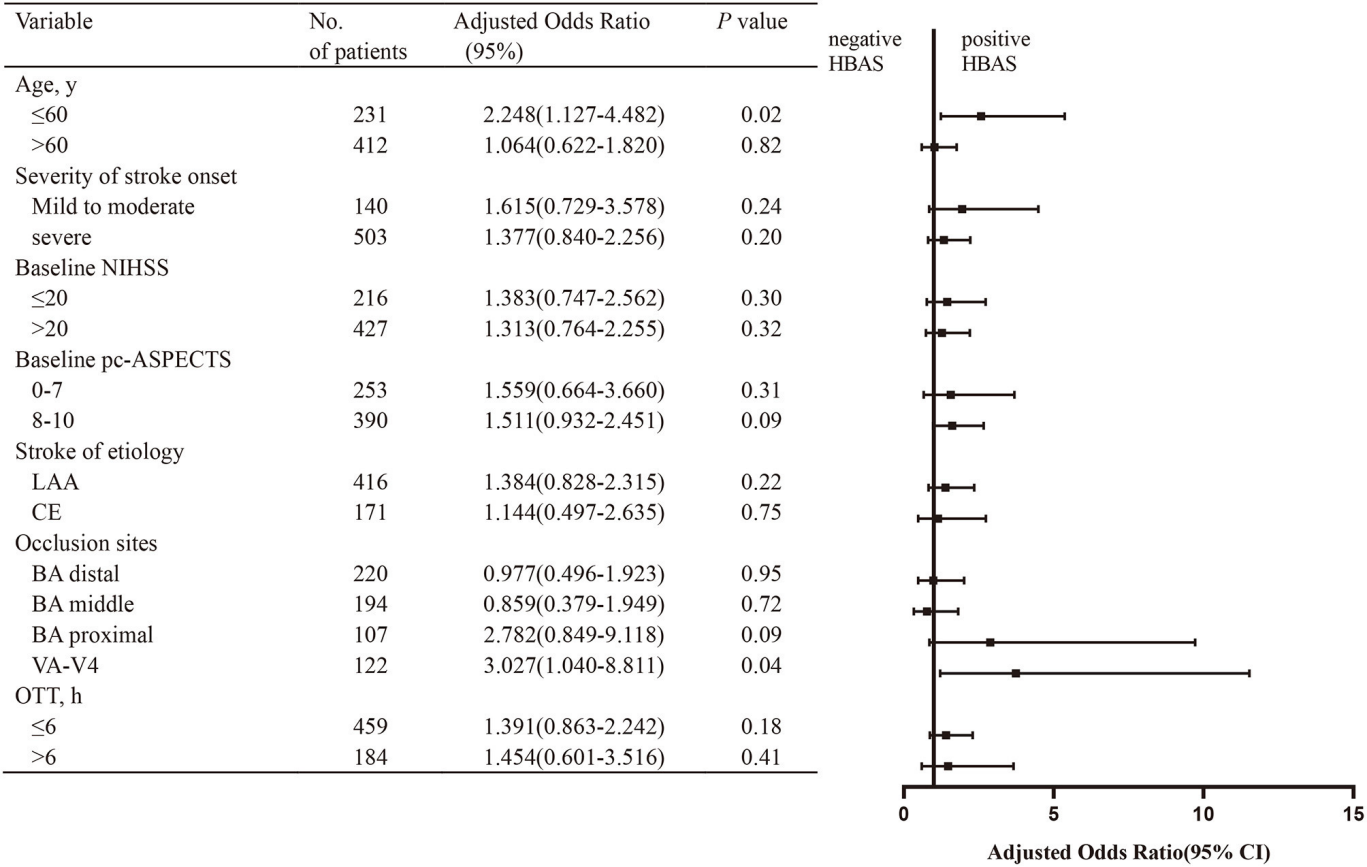
Subgroup analysis of the effect of HBAS for favorable outcome. The forest plot shows that the association of HBAS with favorable outcome (defined as mRS of 0–3) at 90 days differed among patients with various characteristics. The adjusted odds ratio (aOR) was calculated by using binary logistic regression, taking the following variables into account: age, hypertension, diabetes, baseline National Institutes of Health Stroke Scale (NIHSS), baseline posterior circulation–Acute Stroke Prognosis Early Computed Tomography Score (pc-ASPECTS), stroke of etiology, occlusion sites, onset to treatment time (OTT), and American Society of Interventional and Therapeutic Neuroradiology/Society of Interventional Radiology (ASITN/SIR). NIHSS, National Institutes of Health Stroke Scale; pc-ASPECTs, Posterior Circulation-Alberta Stroke Program Early; ASITN/SIR, American Society of interventional and Therapeutic Neuroradiology/Society of interventional Radiology System, LAA, large artery atherosclerosis; CE, cardioembolism; BA, basilar artery; VA-V4, vertebral artery-V4 segment; OTT, onset to treatment time.

As shown in Figure 4, in patients aged 60 years or younger, HBAS (aOR: 2.574, 95% CI: 1.234–5.368; *p* = 0.01) was associated with favorable outcomes after EVT. The association of HBAS with favorable outcomes was also significant in patients with VA-V4 occlusion (aOR: 3.738, 95% CI: 1.212–11.530; *p* = 0.02). For the other subgroups, the relationship between HBAS and favorable outcomes was not significant.

### Predictors of Favorable Outcomes and Successful Recanalization in Patients With HBAS

The predictors of favorable outcomes and successful recanalization were also analyzed in patients with HBAS (Table 3). The admission NIHSS score, distal BAO (aOR: 2.227; 95% CI: 1.163–4.262; *p* = 0.02), ASITN/SIR, recanalization, and puncture to recanalization time were independent predictors of favorable outcomes. Admission NIHSS, pc-ASPECTS, first-pass effect (aOR: 3.573, 95% CI: 1.698–7.521; *p* = 0.001), and stent retriever use (aOR: 2.500, 95% CI: 1.264–4.944; *p* = 0.008) were independent predictors of successful recanalization.

**Table 3:** Multivariate analysis: predictors of favorable outcome and successful recanalization (mTICI 2b to c) in patients with hyperdense basilar artery sign (HBAS) after endovascular treatment (EVT).

**Variables**	**Favorable Outcome^†^**	
	**Adjusted OR**	***P*** **-value**
Age	0.969(0.944–0.995)	0.02
Baseline NIHSS score	0.914(0.883–0.947)	<0.001
Baseline pc-ASPECTS	1.605(1.290–1.996)	<0.001
ASITN/SIR	1.634(1.143–2.335)	0.007
Successful recanalization	4.649(1.671–12.932)	0.003
BA distal occlusion	2.227(1.163–4.262)	0.02
Puncture to recanalization time	0.995(0.990-1.001)	0.09
	**mTICI(2b-c)^‡^**
	**Adjusted OR**	***P*** **-value**
Age	1.015(0.986–1.044)	0.32
Baseline NIHSS score	0.956(0.922–0.991)	0.01
Baseline pc-ASPECTS	1.235(1.030–1.481)	0.02
**Stroke of etiology**		
LAA	Reference	0.12
CE	1.771(0.585–5.365)	0.31
Other	0.734(0.212–2.536)	0.63
**Occlusion sites**		
BA distal	Reference	0.19
BA middle	1.617(0.566–4.622)	0.37
BA proximal	0.620(0.251–1.529)	0.30
VA-V4	0.931(0.337–2.571)	0.89
First pass effect	3.573(1.698–7.521)	0.001
Stent retriever use	2.500(1.264–4.944)	0.008
Puncture to recanalization time	0.996(0.991–1.001)	0.13

† *Favorable outcome was estimated using multiple regression taking those variables into account: age, baseline NIHSS, baseline pc-ASPECTS, diabetes, successful recanalization, ASITN/SIR, first pass, puncture to recanalization time, distal occlusion*.

‡ *Successful recanalization was estimated taking those variables into account: age, baseline NIHSS, baseline pc-ASPECTS, hypertension, stroke of etiology, occlusion sites, first pass, stent retriever use, puncture to recanalization*.

*NIHSS, National Institutes of Health Stroke Scale; pc-ASPECTS, Posterior circulation Acute Stroke Prognosis Early Computed Tomography Score; ASITN/SIR, American Society of interventional and Therapeutic Neuroradiology/Society of interventional Radiology System; mTICI, modified Thrombolysis in Cerebral Infarction*.

## Discussion

Our study showed that there were no significant differences between the HBAS and negative HBAS groups in the rates of favorable outcomes, successful recanalization, and mortality after EVT. However, favorable outcomes were associated with HBAS after EVT in patients below 60 years of age or in those with VA-V4 occlusion.

Studies on anterior and posterior circulation ischemic stroke demonstrated that the HAS on non-enhanced CT was associated with a greater likelihood of stroke severity and poorer clinical outcomes (5, 6, 14). For acute BAO, we found that 51.32% of patients had HBAS on non-enhanced CT, which was accompanied with higher NIHSS scores (median 28) and more severe neurological deficits on admission (83.3%). The presence of severe neurological deficits in the HBAS group is in line with the existing literature (5, 21). The possible reason was the histological characteristics of thrombi that presented with HAS, since they were more likely to be erythrocyte cell-predominant clots, whereas thrombi without HAS were more likely to be fibrin/platelet-predominant clots (8–10). Considering this histological characteristic, erythrocyte-predominant thrombi are a conglomerate of tightly packed erythrocytes that impede blood flow and contrast agent penetration, resulting in poor to absent tissue oxygenation distally (22). Furthermore, atrial fibrillation was considered the main cardiovascular risk factor for acute artery occlusion and was significantly more frequent in patients with HAS (65.3 vs. 45.7%) in anterior circulation stroke, as reported by Kim et al. (23). This result was consistent with our result, where the proportion of atrial fibrillation was higher in the HBAS group than in the negative HBAS group (25.5 vs. 16.6%). Our data showed that the elderly (age ≥ 65 years) were more common in the HBAS group 54.5 vs. 44.1%).

Prior clinical studies have investigated the association of HAS with successful recanalization after EVT. Particularly, some studies indicated that a hyperdense vessel sign was associated with higher rates of recanalization after thrombectomy using the Merci (hyperdense [79%] vs. isodense [36%], *p* = 0.001) or Solitaire retriever (no recanalization vs. recanalization, Hounsfield unit 43.8 ± 6.6 vs. 49.9 ± 7.6, respectively, *p* = 0.01) in the anterior circulation (11, 12). They speculated that the composition consisting predominantly of erythrocytes increased the deformability and viscoelasticity of the thrombi when compared to a composition consisting predominantly of fibrin/platelets (24). Conversely, the fibrin/platelet-predominant thrombi could not be easily penetrated during EVT due to their rigidity and low deformability. However, the association between HBAS and successful recanalization in our study was not significant. The discrepancy might be due to proficient procedural skills, which were effective in removing red and white thrombi. Indeed, successful recanalization rates in our study (81.8%) were much higher than that reported in previous studies (36%) using the Merci device (11) and Solitaire retriever (59%) (12). The first pass effect was achieved more frequently in our study (HBAS [39.1%] vs. negative HBAS [42.2%]) compared to the results from a prior study (HAS [37.63%] vs. non-HAS [35.29%]) by Mohammaden et al. (13) He et al. (25) supported that the possibility of HAS predicting recanalization was low in patients with large artery occlusion undergoing EVT. Particularly, in patients with acute BAO, Shu et al. also support our results that the thrombus density was not different between patients with and without successful recanalization (16).

Recently, Ume et al. reported that the hyperdense middle artery sign was associated with favorable outcomes and lower mortality using modern mechanical devices (26). In our study, favorable outcomes and mortality were not significantly different for patients with or without HBAS after EVT. The reason could be that the HBAS group had more severe stroke deficits, with a higher NIHSS score compared to the negative HBAS group (median 28 vs. 25), which is a well-known risk factor for a worse prognosis. Secondly, successful recanalization is a well-known predictor of favorable outcomes; however, the rate of successful recanalization was not different in our study. In addition, the different anatomical structures might explain the difference in the anterior and posterior circulation. In the current study, clinical outcomes at 90 days were worse in patients with posterior circulation ischemic stroke than in those with the anterior circulation type (27). Similar to our results, Spiotta et al. (28) and Kim et al. (23). reported that HAS was not significant in predicting the clinical outcomes after EVT.

Notably, in patients aged 60 years or younger, our data showed that HBAS was an independent predictor of a favorable outcome. We speculated that it might be due to treatment bias by neurologists because the time from door to puncture was obviously shorter (mean 169.414 vs. 198.118 min) in patients with HBAS, which was also reported in other studies [Froehler et al. (11) and Kim et al. (23)]. Moreover, evidence had showed that door-to-puncture time was an important marker in the delivery of EVT (29), and more rapid workflow was associated with better functional outcomes. In addition, the pooled results showed that diabetes and hyperlipidemia were causal comorbidities that exacerbated ischemic brain injury and worsened functional outcomes after stroke, which might have resulted from the impaired integrity of the large and small blood vessels (30, 31). In our study, we found a lower rate of diabetes (17 vs. 25.2%) and hyperlipidemia (38 vs. 44.3%) in the HBAS cohort than in the cohort without HBAS, although the differences were not statistically significant. Another finding was that HBAS was associated with a favorable outcome after EVT for VA-V4 occlusion. The favorable outcome of the HBAS cohort was 3.585 times higher than that of the negative HBAS cohort. Previous studies have demonstrated that good collateral arterial network (32) and bridging treatment (33) were independently associated with lower mortality and better functional independence in BAO cohorts with VA-V4 occlusion. Meanwhile, our data showed that patients with HBAS had a larger collateral arterial network (22.2 vs. 10.3%) and higher bridging treatment rates (22.2 vs. 13.2%), although the statistical significance was marginal due to the moderate sample size.

Our study has some limitations. First, the BASILAR study had the inherent weakness of non-randomized clinical trials. The best design for preventing bias is the randomized controlled trial. Second, some measures related to HBAS, such as the Hounsfield unit and the length or volume of the HBAS, were not estimated in our study. The Hounsfield unit data were unavailable because the device and imaging technicians were different in the 47 comprehensive stroke centers. However, imaging data were assessed using central assessment, which reduced the bias. Third, the HAS was obtained from 3 to 5 mm-thick slices of non-enhanced CT images; a small clot density might be neglected owing to the greater slice thickness. Thus, the slice should be carefully scanned for accurate clot density. Fourth, the EVT strategy was chosen and applied inconsistently (stent retriever, 75.0%; aspiration, 3.1%; balloon angioplasty and/or stenting, 10.2%; intra-arterial medication and/or mechanical fragmentation, 11.7%). Overall, our study is representative of patients with HBAS in BAO in the mechanical thrombectomy era, despite its limitations.

## Conclusion

HBAS is not an independent predictor of favorable outcomes and successful recanalization in patients with BAO who underwent EVT. However, large-scale, prospective national, or even international, registry data are needed to elucidate the association of HBAS with successful recanalization and favorable outcomes in patients with BAO.

## Data Availability Statement

The original contributions presented in the study are included in the article/Supplementary Material, further inquiries can be directed to the corresponding author/s.

## Ethics Statement

The studies involving human participants were reviewed and approved by the Ethics Committee of the Xinqiao Hospital, Army Medical University, in Chongqing, China, and each subcenter (No 201308701). The patients/participants provided their written informed consent to participate in this study.

## Author Contributions

QY and CL made substantial contributions to the conception or design of the work. JH, WH, and BZ made contributions to analysis and interpretation of data in the work. JH, FH, and KL drafted the work or revised it critically for important intellectual content. JiasL, ZC, HJ, KW, HW, YL, and JiacL approved the version to be published. WZ, FL, HS, SL, WL, RS, JY, JH, and JS agree to be accountable for all aspects of the work in ensuring that questions related to the accuracy or integrity of any part of the work are appropriately investigated and resolved. All authors contributed to the article and approved the submitted version.

## Funding

This study was supported by the National Natural Science Foundation of China (No. 82071323), Chongqing Natural Science Foundation (cstc2020jcyj-msxmX0926), Chongqing Health Appropriate Technology Promotion Project (No. 2020jstg038), Chongqing Science and Health Joint Project (No. 2019ZDXM002), and Clinical Medical Research Personnel Training Program (No. 2018XLC1005).

## Conflict of Interest

The authors declare that the research was conducted in the absence of any commercial or financial relationships that could be construed as a potential conflict of interest.

## Publisher's Note

All claims expressed in this article are solely those of the authors and do not necessarily represent those of their affiliated organizations, or those of the publisher, the editors and the reviewers. Any product that may be evaluated in this article, or claim that may be made by its manufacturer, is not guaranteed or endorsed by the publisher.
