# Supplementary material for: Hyperdense Artery Sign and Clinical Outcomes After Endovascular Treatment in Acute Basilar Artery Occlusion
Source: Front Neurol. 2022 Apr 25;13:830705. doi: 10.3389/fneur.2022.830705 (PMC9081764; doi:10.3389/fneur.2022.830705)
Supplement: Supplementary file 1 [file Data_Sheet_1.docx]

**Supplementary Appendix**

This appendix has been provided by the authors to give readers additional information about their work

**eTable 1.** Univariate analysis of favorable outcome

**eTable 2.** Univariate analysis of successful recanalization

**eTable 3.** Time characteristic and cardiovascular risk factors of patients younger than 60 underwent EVT

**eTable 4.** Bridging treatment rates and collateral status for VA-V4 occlusion

eTable1. Univariate analysis of favorable outcome

|  | Favorable  outcome | Unfavorable outcome | P value |
| --- | --- | --- | --- |
| Age, median (IQR), y | 64(55-72) | 66(60-74) | 0.09 |
| Male, n (%) | 81(76.4%) | 170(75.9%) | 0.92 |
| baseline NIHSS, median (IQR) | 20(12-27) | 30(23-34) | <0.001 |
| Neurology deficit, n (%) |  |  | <0.001 |
| Mild to moderate | 32(30.2%) | 23(10.3%) |  |
| Severe | 74(69.8%) | 201(89.7%) |  |
| pc-ASPECTS, median (IQR) | 9(8-10) | 7(6-9) | <0.001 |
| Hypertension, n (%) | 71(67.0%) | 160(71.4%) | 0.41 |
| Diabetes, n (%) | 18(17%) | 57(25.4%) | 0.09 |
| Pre-stroke, n (%) | 23(21.7%) | 56(25%) | 0.51 |
| Atrial fibrillation, n (%) | 29(27.4%) | 55(24.6%) | 0.59 |
| Etiology of stroke, n (%) |  |  | 0.29 |
| LAA | 60(56.6%) | 143(63.8%) |  |
| CE | 33(31.1%) | 64(28.6%) |  |
| other | 13(12.3%) | 17(7.6%) |  |
| Occlusion sites, n (%) |  |  | 0.39 |
| Distal basilar artery | 43(40.6%) | 76(33.9%) |  |
| Middle basilar artery | 26(24.5%) | 75(33.5%) |  |
| Proximal basilar artery | 18(17%) | 38(17%) |  |
| VA-V4 | 19(17.9%) | 35(15.6%) |  |
| Recanalization, n (%) | 100(94.3%) | 163(72.8%) | <0.001 |
| ASITN/SIR, median (IQR) | 2(1-2) | 1(0-2) | <0.001 |
| First pass, n (%) | 51(48.1%) | 78(34.8%) | 0.02 |
| Onset to puncture time, median (IQR), min | 306.5(206.75-445.75) | 340(234-496) | 0.24 |
| Puncture to recanalization time, median (IQR), min | 87(60-135.75) | 109(78-160) | 0.003 |

Abbreviations: NIHSS, National Institutes of Health Stroke Scale; pc-ASPECT, posterior circulation Acute Stroke Prognosis Early Computed Tomography Score; LAA, large artery atherosclerosis; CE, cardioembolic; VA-V4, vertebral artery-V4 segment; ASITN/SIR, American Society of interventional and Therapeutic Neuroradiology/Society of interventional Radiology System.

eTable2. Univariate analysis of successful recanalization

|  | mTICI (0-2a) | mTICI (2b-3) | *P* value |
| --- | --- | --- | --- |
| Age, median (IQR), y | 65(55-74) | 66(59-74) | 0.47 |
| Male, n (%) | 47(70.1) | 204(77.6) | 0.20 |
| Baseline NIHSS, median (IQR) | 32(23-35) | 27(17-34) | 0.01 |
| Neurology deficit, n (%) |  |  | 0.02 |
| Mild to moderate | 5(7.5) | 50(19.0) |  |
| Severe | 62(92.5) | 213(81.0) |  |
| pc-ASPECT, median (IQR) | 7(6-8) | 8(7-9) | 0.003 |
| Hypertension, n (%) | 48(71.6) | 183(69.6) | 0.74 |
| Diabetes, n (%) | 17(25.4) | 58(22.1) | 0.56 |
| Pre-stroke, n (%) | 18(26.9) | 61(23.2) | 0.53 |
| Atrial fibrillation, n (%) | 16(23.9) | 68(25.9) | 0.74 |
| Etiology of stroke, n (%) |  |  | 0.64 |
| LAA | 39(58.2) | 164(62.4) |  |
| CE | 20(29.9) | 77(29.3) |  |
| other | 8(11.9) | 22(8.4) |  |
| Occlusion sites, n (%) |  |  | 0.54 |
| Distal basilar artery | 20(29.9) | 99(37.6) |  |
| Middle basilar artery | 25(37.3) | 76(28.9) |  |
| Proximal basilar artery | 11(16.4) | 45(17.1) |  |
| VA-V4 | 11(16.4) | 43(16.3) |  |
| ASITN/SIR, median (IQR) | 1(0-2) | 1(1-2) | 0.06 |
| Stent retriever use, n (%) | 42(62.7) | 203(77.2) | 0.02 |
| First pass, n (%) | 10(14.9) | 119(45.2) | <0.001 |
| Onset to puncture time, median (IQR), min | 344(232-494) | 328.5(217.5-484.25) | 0.69 |
| Puncture to recanalization time, median (IQR), min | 125(85-164) | 101(68.75-151) | 0.01 |

Abbreviations: mTICI, modified Thrombolysis in Cerebral Infarction; NIHSS, National Institutes of Health Stroke Scale; pc-ASPECT, posterior circulation Acute Stroke Prognosis Early Computed Tomography Score; LAA, large artery atherosclerosis; CE, cardioembolic; VA-V4, vertebral artery-V4 segment; ASITN/SIR, American Society of interventional and Therapeutic Neuroradiology/Society of interventional Radiology System.

eTable3. Time characteristic and cardiovascular risk factors of patients younger than 60 underwent EVT

|  | Negative HBAS | Positive HBAS | P value |
| --- | --- | --- | --- |
| Diabetes, n (%) | 33(25.2) | 17(17) | 0.13 |
| Hypertension, n (%) | 81(61.8) | 62(62) | 0.98 |
| Hyperlipidemia, n (%) | 58(44.3) | 38(38) | 0.34 |
| Smoking, n (%) | 62(47.3) | 47(47) | 0.96 |
| Door to puncture(mean) | 198.118 | 169.414 | 0.27 |
| Door to recanalization (mean) | 308.828 | 285.044 | 0.57 |

Abbreviations: EVT, endovascular treatment; HBAS, hyperdense basilar artery sign.

eTable4. Bridging treatment rates and collateral status for VA-V4 occlusion

|  | Negative HBAS | Positive HBAS | P value |
| --- | --- | --- | --- |
| ASITN/SIR (mean) | 1.28 | 1.37 | 0.87 |
| ASITN/SIR, n (%) |  |  | 0.051 |
| 0-1 | 42(61.8) | 35(64.8) |  |
| 2 | 19(27.9) | 7(13.0) |  |
| 3-4 | 7(10.3) | 12(22.2) |  |
| Bridging therapy, n (%) | 9(13.2) | 12(22.2) | 0.192 |

Abbreviations: VA-V4, vertebral artery-V4 segment; EVT, endovascular treatment; HBAS, hyperdense basilar artery sign; ASITN/SIR, American Society of interventional and Therapeutic Neuroradiology/Society of interventional Radiology System.
